# Comparison of Risk Factors for Pediatric Kidney Stone Formation: The Effects of Sex

**DOI:** 10.3389/fped.2019.00032

**Published:** 2019-02-12

**Authors:** Andrew L. Schwaderer, Rupesh Raina, Anshika Khare, Fayez Safadi, Sharon M. Moe, Kirsten Kusumi

**Affiliations:** ^1^Division of Nephrology, Department of Pediatrics, Indiana University School of Medicine, Indianapolis, IN, United States; ^2^Division of Nephrology, Akron Children's Hospital, Akron, OH, United States; ^3^Northeast Ohio Medical University, Rootstown, OH, United States; ^4^Division of Nephrology, Department of Medicine, Indiana University School of Medicine, Indianapolis, IN, United States

**Keywords:** urolithiasis, kidney stones, pediatrics, sex, age

## Abstract

**Background:** Urinary stones are affecting more children, and pediatric stone formers have unique pathophysiology compared to adults. While adult stone formers are most frequently male, children have an age dependent sex prevalence. Under 10 years, a majority of stone formers are boys; adolescent stone formers are mostly female. Previous adult studies have shown that stone composition is influenced by the sex and age of the stone former. Thus, we hypothesize that female and male stone forming children will also have sex and age specific stone phenotypes.

**Methods:** Retrospective chart review of a large pediatric center's stone forming children 6/1/2009 to 6/1/2016. Patients were identified by ICD 9 codes: N20, N20.1, and N20.9. Charts were reviewed for radiographic evidence of stones or documented visualized stone passage.

**Results:** One hundred and thirty six subjects: 54 males and 82 females. Females were older, median age 14 years [interquartile range (IQR): 11, 15] vs. males' median age 12 years (IQR: 11, 14) (*p* < 0.01). Females had lower height *z*-scores, median 0.2 (IQR: −0.8, 0.8) vs. males' median 0.8 (IQR: −0.2, 1.8) (*p* < 0.01). Presenting symptoms were similar except flank pain affecting 39% of females vs. 22% of males (*p* = 0.04). Leukocyte esterase was positive in more females than males (33 vs. 4%) (*p* < 0.001). Males had a higher BUN/Cr ratio, mean ± standard deviation of 19.8 ± 6.3 vs. 16.6 ± 6.5 in females (*p* = 0.01). Glomerular hyperfiltration was present in 9% of patients while 35% of patients had estimated glomerular filtration rate (eGFR) < 90 ml/min/1.73 m^2^. Treatment strategies and clinical course were similar except females were told to increase dietary citrate more frequently than males (21 vs. 4%) (*p* < 0.01).

**Conclusion:** We have provided a novel analysis and demonstrated that low height *z*-score and pyuria are more common in female stone formers. We have also shown that 9% of pediatric stone formers have labs consistent with hyperfiltration. Whether high protein intake and/or chronic dehydration are associated with hyperfiltration and long-term renal function in children with kidney stones will be an area for future research.

## Introduction

Urinary stone disease is a frequent cause of morbidity in the United States with approximately 10% of adults developing a stone in their lifetime, and half of those affected will have recurrent stones (1). Traditionally, urinary stones were considered an adult disease. However, there is mounting evidence for increased stone formation in the pediatric population, and the number of children seen for urinary stone disease doubled between 1997 and 2012 (2, 3). The monetary burden of stone disease is also growing and adult stone care charges are now 10 billion dollars annually (4). Hospital costs/charges for stone forming children are similarly increasing, and inpatient care in 2012 totaled $17.5 million, a 20% increase over 15 years after adjusting for health care inflation and population growth (2). The mechanisms driving increased stone formation in children remains unknown; however, urinary stones now account for 1 in 685 pediatric admissions (5).

The adage that “children are not little adults” may be applied to pediatric stone formers as they have a unique stone phenotype compared to adults. Children are more likely than adults to have specific metabolic derangements driving their stone formation and also have lower rates of spontaneous stone passage (6, 7). As a result children generally undergo a more comprehensive metabolic work up compared to adults and are more likely to require surgical intervention for their acute stone treatment (8). In addition, pediatric stone formers have distinctive sex prevalence patterns. While adults with stones are most frequently male, pediatric stone formers have an age dependent sex prevalence (1, 2). Novak et al. (8) demonstrated that stone forming children <10 years of age are more often male. However, in the adolescent age range, girls form more stones than boys (2, 8). Adolescent females also represent the largest proportion of the pediatric stone forming population overall (2, 8). This finding of age dependent sex prevalence and preponderance of adolescent girls in the pediatric urinary stone population have been replicated by other groups using the Healthcare Cost and Utilization Project Kids' Inpatient Database (2, 9). Furthermore, adult stone formers have stone phenotypes that are influenced by sex and age (10). Despite these studies, we have very limited data describing stone risk factors, presentation, and management differences between pediatric male and female stone patients. Therefore, this retrospective analysis was performed to evaluate a pediatric stone forming cohort for lithogenic risk factors and descriptive patient characteristics while divided by sex. The objective of this study was to define the urinary stone patient epidemiology specific to female and male patients who were evaluated at a single tertiary care children's hospital.

## Materials and Methods

This is a retrospective review of all urinary stone patients seen at Akron Children's Hospital during a 7-year time period (June 2009 to June 2016) and was approved by the Akron Children's Institutional Review board (1040501-5). All children and adolescents ≤19 years of age who were treated for urinary stones at Akron Children's hospital were included. Patients were identified by the following diagnostic codes: ICD 10: N20 (calculus of kidney), N20.1 (calculus of ureter), and N20.9 (urinary calculus, unspecified) and ICD9: 592 (calculus of kidney and ureter), 592.1 (calculus of ureter), 592.9 (urinary calculus, unspecified). After identification by ICD9 code, individual charts were reviewed and patients without radiographic evidence of stones or visualized stone passage confirmed by attending physician documentation were excluded. The clinical data obtained included: age, sex, height, weight (all recorded from initial stone presentation), associated medical diagnoses, history of skeletal fractures, medications, presenting symptoms, number of stones, number of stone surgeries, number of emergency room visits and hospital admissions related to urinary stones, number and type of imaging related to urinary stones, requirement of subspecialty care, medical interventions for stone, family history of stones, serum chemistries, stone analysis, vitamin D, PTH, urine studies (pH, specific gravity, blood, protein), 24 h urine studies (urine Na, Ca, Ox, Phos, citrate), and stone analysis. The height, weight and BMI were converted to *z*-scores by using CDC defined normative values (https://www.cdc.gov/growthcharts/percentile_data_files.htm). Subjects were then compared by sex, females vs. males. Estimated glomerular filtration rate (eGFR) was determined using the bedside Schwartz equation (11).

Data examination included calculation of full summary statistics for continuous variables, as well as calculations of frequencies and percentages for categorical variables. Continuous data was evaluated for normality using the D'Agostino and Pearson normality test. If data from both groups were normally distributed the Welch's *t*-test was used, if at least one group had data that was not normally distributed the Mann-Whitney test was used. For percentages, the Pearson Chi-square test was used only if all excepted cell frequencies were ≥5, otherwise the Fisher exact probability test was used. Graphpad PRISM was used to analyze continuous data and perform linear regression while VassarStats (http://vassarstats.net/) was used to compare percentages (12). All testing was two- tailed and evaluated at the Type I error rate of alpha = 0.05 level of statistical significance.

## Results

### Patients

A total of 185 individual subjects were identified through ICD 9 diagnostic codes. Of these, 49 did not have radiographic evidence of urinary stones and/or documented stone passage on chart review and were excluded. Therefore, a total of 136 subjects were analyzed in this study; these were subdivided into 54 males and 82 females.

### Presentation

Patient presentation is detailed in Table 1. Female patients were significantly older with median age 14 years (interquartile range (IQR: 11, 15) vs. males with median 12 years (IQR: 11, 14) (*p* < 0.01). Females also had lower height *z*-scores with median 0.2 (IQR: −0.8, 0.8) compared to males with median 0.8 (IQR: −0.2, 1.8) (*p* < 0.01). Five patients were identified with congenital anomalies of the kidney and urinary tract including: horseshoe kidney, neurogenic bladder with Mitrofanoff, duplex kidney, congenital renal dysplasia, and medullary sponge kidney. Twenty-nine patients had dilation of the renal collecting system on imaging. One patient was diagnosed with Renal tubular acidosis (RTA) and one patient had cystinuria. No other monogenic or genetic stone diagnoses were present nor did any patients have formally diagnosed Rickets. Of note, two patients had Crohn's disease and one had Ulcerative Colitis. Presenting symptoms were similar between the groups except for flank pain which was described by 39% of female patients vs. only 22% of male patients (*p* = 0.04). Male and female stone patients had similar and very low rates of Furosemide and Topiramate exposure (4 females and 2 males both accounting for 4% of their respective populations). Both females and males had near identical rates of reporting a family history of urinary stones (65 and 64%, respectively) despite low rates of monogenic stone disease.

**Table 1:** Presentation.

	**Female (*****n*** **=** **82)**		**Male (*****n*** **=** **54)**		**Statistical test**	***P*-value**
	**Mean ± Std Dev**	**Median (25, 75%)**	**Mean ± Std Dev**	**Median (25, 75%)**		
Age (years)	12.9 ± 2.8	14 (11,15)	11.7 ± 2.8	12 (11,14)	Mann- Whitney	* < 0.01
^∧^Height z-score	−0.25 ± 1.80	0.17 (−0.8, 0.8)	0.73 ± 1.34	0.83 (−0.2, 1.8)	Mann-Whitney	* < 0.01
^∧^Weight z-score	0.67 ± 1.43	1.0 (−0.2, 1.7)	0.96 ± 1.61	0.9 (−0.5, 2.4)	Mann- Whitney	0.60
^∧^BMI z-score	0.91 ± 1.16	1.1 (1.2, 1.8)	0.50 ± 1.60	0.3 (−1.0, 2.2)	*t*-test	0.25
	**Number**	**%**	**Number**	**%**		
Hematuria	12	15	9	17	Chi-square	0.75
Flank pain	32	39	12	22	Chi-square	* 0.04
Abd pain	44	54	32	59	Chi-square	0.52
Nausea	29	35	18	33	Chi square	0.80
Vomiting	28	34	12	22	Chi square	0.11
Fever	5	6	5	9	Fisher exact	0.52
Dysuria	8	10	6	11	Chi square	0.76
Back pain	12	15	7	13	Chi square	0.78
Dilated collecting system^#^	21	25	8	15	Chi square	0.13
Furosemide	2	2	2	4	Fisher exact	1.00
Topiramate	2	2	0	0	Fisher exact	0.52
Fracture hx without comorbidities	7	8.5	4	7.4	Chi square	0.82
Family hx of urinary stones	53	65	35	64	Chi square	1.00

∧ *The n for height, weight and BMI z-score was 49 females and 27 males*,

# *hydronephrosis or pelvocaliectasis on CT or US*.

* *Significant p-values are indicated in Bold*.

### Evaluation

The evaluation of urinary stone patients (Table 2) was similar between male and female patients. Of note, CTs were obtained more than twice as often as renal ultrasound (RUS) in both male and female urinary stone formers; females: 67% CT vs. 28% RUS and males: 57% CT vs. 20% RUS. Serum basic metabolic panels (BMP) and calcium levels were obtained in approximately 75% of patients but serum phosphorous, uric acid, PTH and vitamin D levels were obtained in <25% of both males and females. Twenty-four hour urine stone risk panels (Litholink Corporation, Chicago IL) were obtained in under half of patients and Pediatric Urology referrals were more common than Pediatric Nephrology referrals and only 20% of patients were evaluated by a nephrologist.

**Table 2:** Evaluation.

**Outcome**	**Female (*****n*** **=** **82)**		**Male (*****n*** **=** **54)**		**Statistical test**	***P*-value**
	**Number**	**%**	**Number**	**%**		
CT	55	67	31	57	Chi-square	0.25
US	23	28	11	20	Chi-square	0.21
X-ray	26	32	12	22	Chi-square	0.23
Serum BMP	63	77	44	81	Chi-square	0.51
Serum calcium	60	73	42	78	Chi-square	0.54
Serum phosphorous	15	18	13	24	Chi square	0.41
Serum uric acid	4	5	6	11	Fisher exact	0.19
Serum PTH	2	2	5	9	Fisher exact	0.11
Serum 25 Vitamin D	10	12	13	24	Chi square	0.07
24 h urine stone risk panel (Litholink)	30	37	25	46	Chi square	0.26
Stone analysis	13	16	10	19	Chi square	0.69
Pediatric Urology referral	57	70	37	69	Chi-square	0.89
Pediatric Nephrology referral	14	17	14	26	Chi-square	0.21

### Laboratory Results

Laboratory results for male and female pediatric stone formers are presented in Table 3. Electrolytes were similar between the sexes, but both the BUN and the BUN/Creatinine ratio were higher in male patients. There were no significant differences in 24 h urine levels for calcium, volume, oxalate, citrate, calcium oxalate supersaturation, or calcium phosphate supersaturation. On urinalysis, over 3/4 of both males and females tested positive for hemoglobin and just under 1/2 tested positive for protein. Females had significantly higher rates of positive leukocyte esterase (LE). Twenty-four hour urine stone risk panels (Litholink Corporation, Chicago IL) showed no significant differences between females and males for urinary stone risk factors including citrate, calcium and oxalate levels (13–16). Of 13 urinary stones from females submitted for analysis, 9 (69%) contained calcium oxalate (dihydrate), 7(54%) contained calcium oxalate (monohydrate), 5 (38%) contained calcium phosphate (apatite), 1(8%) contained calcium phosphate (brushite), and 1(8%) contained struvite. Of 10 urinary stones from males submitted for analysis, 7(70%) contained calcium oxalate (dihydrate), 5(50%) contained calcium oxalate (monohydrate), 1(10%) contained ammonium urate, 2(20%) contained calcium phosphate (apatite).

**Table 3:** Lab results.

**Lab**	**Normal**	**Female (*****n*** **= 82)**		***n***	**Male (*****n*** **=** **54)**		***n***	**Statistical test**	***P*-value**
		**Mean ± Std Dev**	**Median (25, 75%)**		**Mean ± Std Dev**	**Median (25, 75%)**			
Serum Na^+^	133–145	138 ± 3	139 (137, 140)	63	139(137, 141)	138 ± 3	44	MW	0.94
Serum K^+^	3.3–5.1	3.9 ± 0.6	3.9 (3.7, 4.1)	63	4.0 ± 0.9	4.0 (3.6, 4.2)	44	MW	0.49
Serum Cl^−^	96–108	105 ± 3	105 (103, 106)	63	105 ± 3	104 (103, 107)	44	*t*-test	0.58
Serum CO_2_	22–29	23.9 ± 2.5	23.9 (22.1, 26.1)	59	24.1 ± 3.0	24.0 (21.8, 26.7)	43	*t*-test	0.77
BUN	4–19	11.1 ± 4.2	10 (9, 13)	63	12.2 ± 2.7	12 (10, 14)	44	MW	*0.02
Creatinine	0.4–1	0.83 ± 1.2	0.7 (0.6, 0.8)	63	0.67 ± 0.22	0.6 (0.5, 0.7)	44	MW	0.19
Serum BUN/creat		16.6 ± 6.5	16 (12, 20)	63	19.8 ± 6.3	19 (16, 24)	44	*t*-test	*0.01
Serum Ca^++^	7.6–11	9.32 ± 0.50	9.3 (9.0, 9.7)	60	9.35 ± 0.46	9.3 (9.0, 9.8)	42	MW	0.79
Serum Phos	2.7–4.5	4.3 ± 0.57	4.4 (4.1, 4.8)	15	4.4 ± 0.51	4.4 (4.1, 4.7)	13	*t*-test	0.77
Serum PTH	15–65	45.5 ± 31.8	45.5 (23, 68)	2	33.8 ± 18.5	34 (17.5, 50)	5	MW	0.57
Serum Vit D	20–50	26.4 ± 9.5	29.5 (18.5, 29.5)	10	28.9 ± 11.6	29 (20, 35)	13	MW	0.79
Uric acid	3–7	6.1 ± 1.7	6 (5, 8)	4	5.2 ± 1.5	5 (4, 7)	6	MW	0.26
Urine volume	>1	0.96 ± 0.49	0.85 (0.58, 1.21)	30	1.02 ± 0.58	0.86 (0.59, 1.29)	25	MW	0.89
Urine Ca^++^	< 4	2.87 ± 1.63	2.6 (1.6, 3.7)	30	3.36 ± 2.02	3.0 (2.1, 4.0)	25	MW	0.41
Urine Cit	>180 m >250 f	622 ± 274	549 (246, 749)	30	562 ± 173	571 (428, 703)	24	*t*-test	0.33
Urine Ox	< 40	33.7 ± 13.3	31.2 (25.0, 40.6)	30	40.3 ± 16.0	38.3 (28.1, 49.3)	24	MW	0.09
CaOx SS	6–10	7.67 ± 3.67	8.3 (5.1, 9.5)	30	8.75 ± 3.64	8.2 (6.5, 10.5)	25	*t*-test	0.28
CaPhos SS	0.5–2	2.11 ± 1.29	2.4 (0.7, 3.1)	30	2.67 ± 1.55	2.5 (1.1, 3.8)	25	MW	0.12
Urine Sul	20–80	30.48 ± 10.65	32.77 (22.75, 37.87)	30	37.18 ± 15.32	35.4 (24.39, 48.57)	24	*t*-test	0.08
UUN	6–14	7.76 ± 2.43	8.03 (6.21, 9.06)	30	9.32 ± 3.73	8.23 (6.81, 11.32)	24	*t*-test	0.09
Urine PCR	8–14	0.97 ± 0.24	0.95 (0.82, 1.12)	30	1.18 ± 0.45	1.08 (0.83, 1.44)	25	MW	0.08
UA: pH	5–7.5	6.3 ± 0.9	6 (6, 7)	64	6.5 ± 1.1	6 (6, 7)	44	MW	0.34
UA: SG	1–1.035	1.019 ± 0.009	1.02 (1.01, 1.03)	66	1.022 ± 0.006	1.02 (1.02, 1.03)	46	*t*-test	0.07
		**Number**	**%**		**Number**	**%**			
UA: +LE	Neg	23	33	69	2	4	46	Fisher exact	* < 0.001
UA: +nit	Neg	2	3	69	0	0	46	Fisher exact	0.52
UA: +hgb	Neg	55	80	69	39	85	46	Fisher exact	0.48
UA: +prot	Neg	33	48	69	20	43	46	Chi square	0.36

Results, Mean ± Std Dev for parametric and Median (25, 75%) for non-parametric data. Serum labs: Na^+^, K^+^, Cl^−^, CO_2_ presented as mEq/L; Ca^++^, Phos, BUN, creatinine, and uric acid presented as mg/dl; Vitamin D in ng/ml; PTH as pg/ml Urine labs: 24 h urine Ca^++^, Citrate, and oxalate presented as mg/kg/1.73 m^2^, Protein catabolic rate (PCR) presented as g/kg/day and urine urea nitrogren (UUN) presented as g/1.73 m^2^/day. Urine sulfates presented as mEq/1.73/m^2^/day UA findings called positive if 1+ or greater. MW, Mann-Whitney. Significant p-values are indicated in Bold. *Significant p-values.

### Metabolic Workup Findings

**Figure 1:**
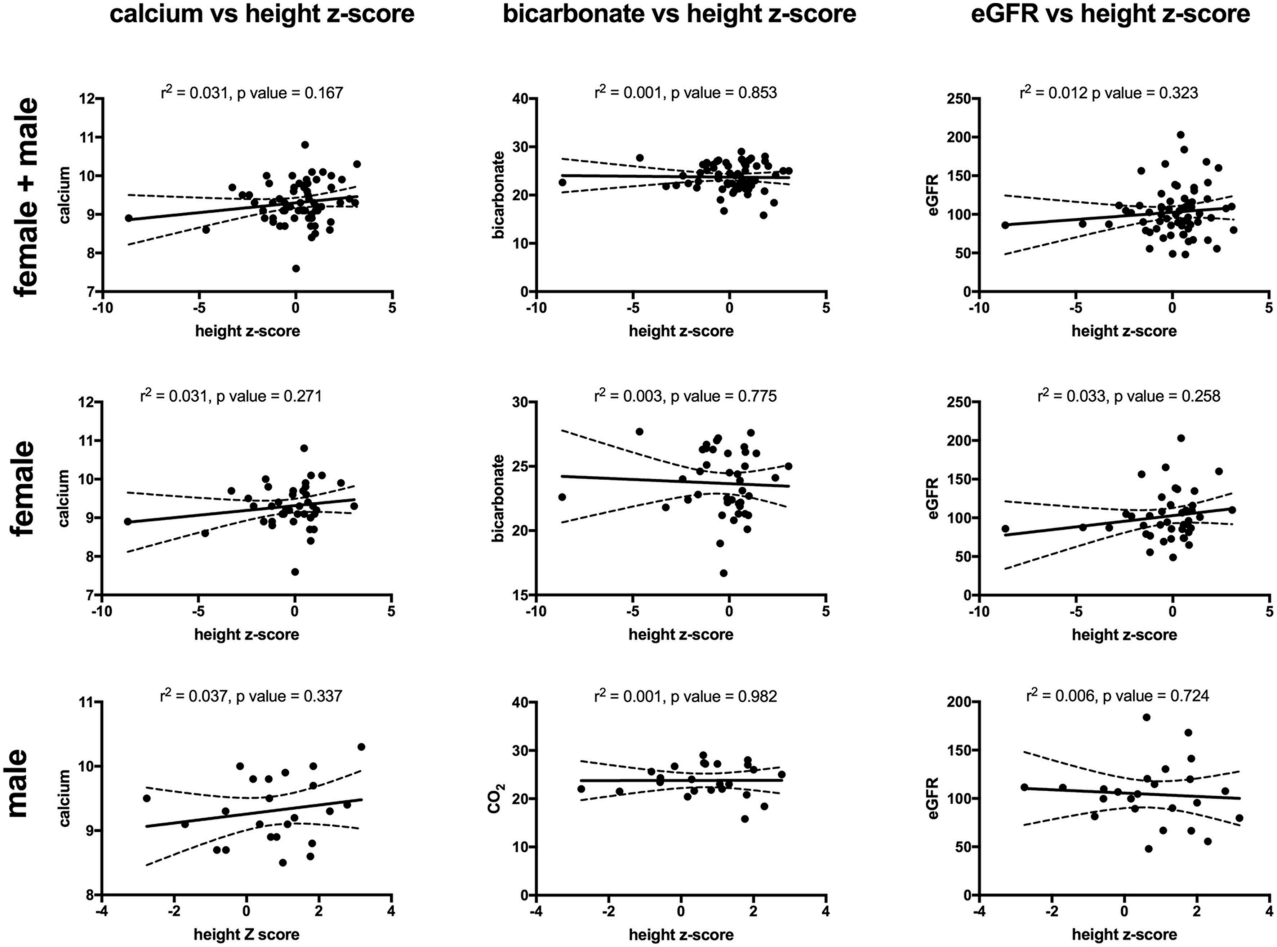
The correlations between height z-score with serum calcium (mg/dl) (left), serum bicarbonate (mEq/L) (middle), and eGFR (ml/min/1.73 m^2^) (right) for all patients (top), females (middle), and males (bottom). There was no significant correlation for any of the comparisons. Sixty-four patients (43 females and 24 males) had both a height *z*-score and serum calcium measured, 65 patients (41 females and 24 males) had both a height *z*-score and serum bicarbonate measured and 65 (41 females and 24 males) patients had both a height *z*-score and eGFR). Serum calcium in mg/dl, eGFR in mil/min/1.73 m^2^ and bicarbonate in mEq/L. The dashed lines represent 95% confidence intervals.

**Figure 2:**
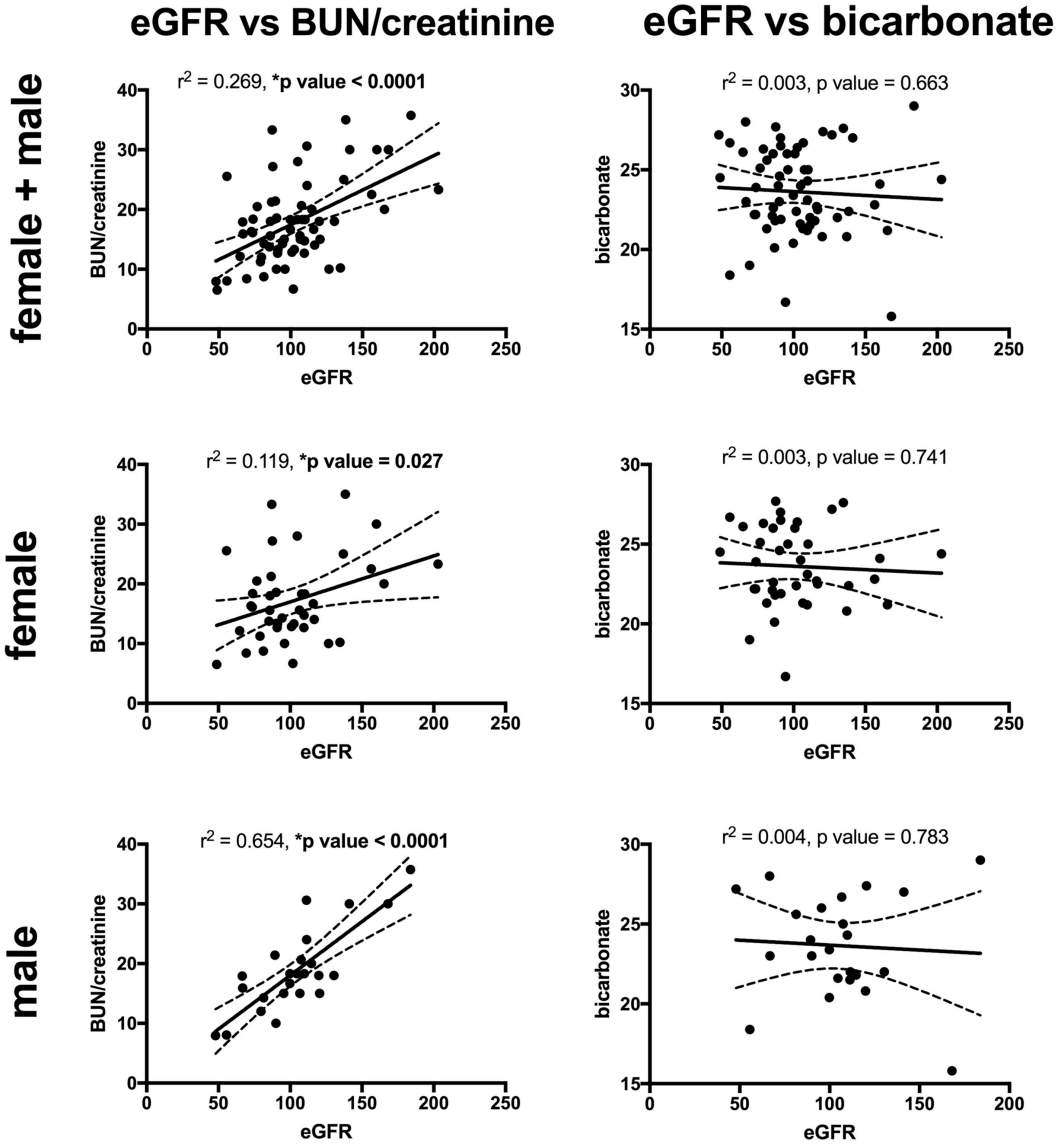
The correlations between eGFR with serum BUN/creatinine ratio (left) and serum bicarbonate (mEq/L) (right), for all patients (top), females (middle), and males (bottom). eGFR had a significant direct correlation with the BUN/creatinine ratio for all patients, females, and males. There was no significant correlation between the eGFR and serum bicarbonate. Sixty-five patients (41 males and 24 females) had both a height *z*-score and serum BUN/creatinine ratio measured and 63 patients (40 females and 23 males) had both an eGFR and serum bicarbonate. Serum BUN/creatinine in mg/mg, eGFR in mil/min/1.73 m^2^ and bicarbonate in mEq/L. The dashed lines represent 95% confidence intervals.

**Figure 3:**
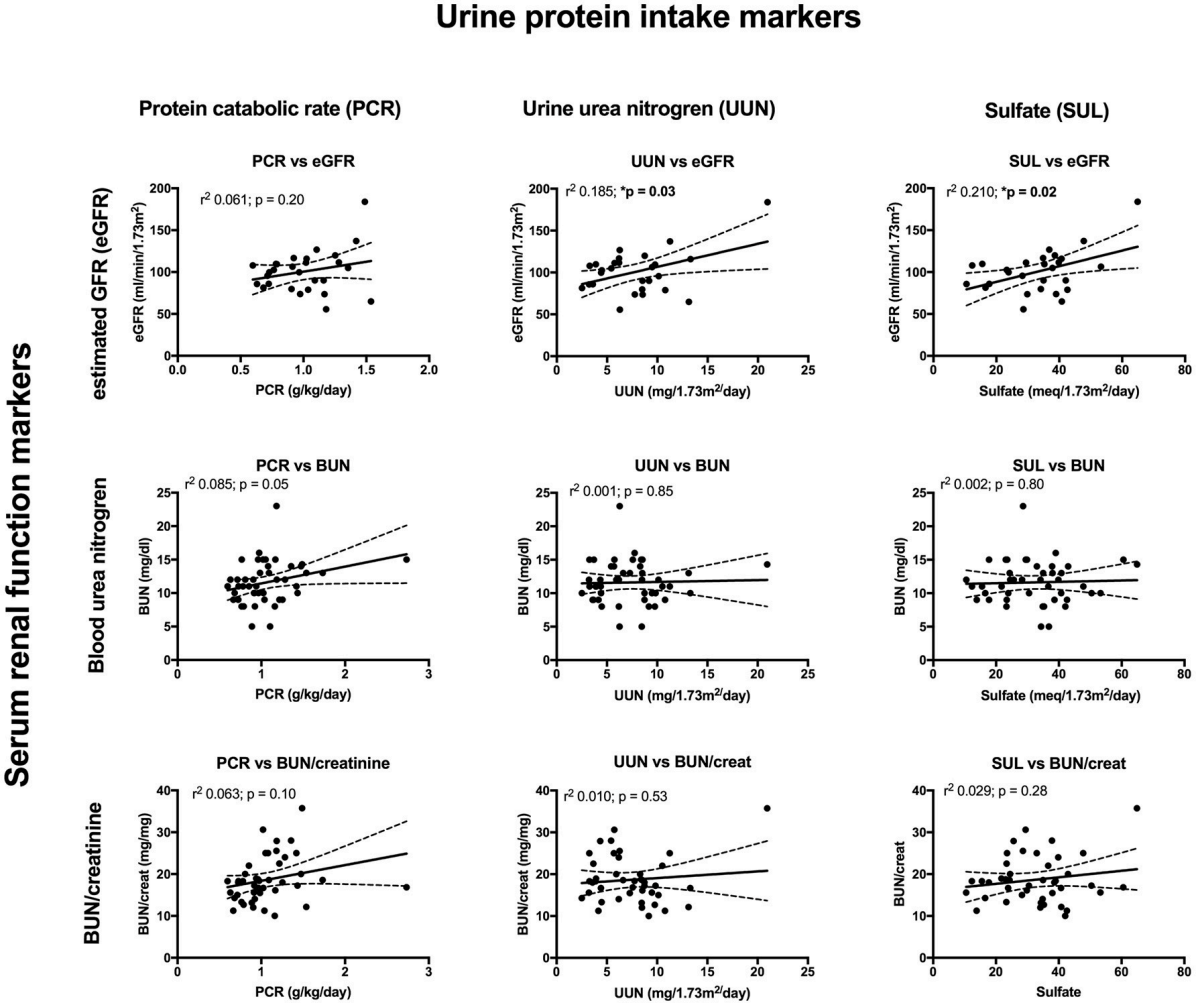
The correlations between urine markers of dietary protein intake consisting of urine protein catabolic rate (PCR) (left), urine urea nitrogen (UUN) (middle), and urine sulfate (SUL) (right), compared to eGFR (top), serum BUN (middle), and serum BUN/creatinine (bottom). The correlation between PCR and BUN just missed significance while there was a significant correlation between UUN and eGFR along with SUL and eGFR.

Key findings on metabolic evaluation are presented in Table 4. Of the 11 females that had a height *z*-score < −1, six had concurrent diagnosis that may explain their short stature including one patient each with Crohn's disease, chronic lymphocytic thyroiditis, “hormone imbalance” and spastic quadriparesis along with 2 patients with chronic kidney disease (CKD). Of the 2 boys with a score of < −1, one had growth hormone deficiency. The most common finding on lab evaluation was low serum bicarbonate (HCO${}_{3}^{-}$or CO_2_) which occurred in 23% of patients. Elevated serum calcium and vitamin D levels were only identified in 1 patient in each group. Glomerular hyperfiltration, defined as an eGFR > 140 ml/min/1.73 m^2^ (17), was present in 9% of patients while 35% of patients had a eGFR < 90 ml/min/1.73 m^2^. Of the 23 children who had an eGFR < 90 ml/min/1.73 m^2^, 10 had concurrent diagnosis that might explain this finding; 4 had known CKD, 3 had pyelonephritis, 2 had acute kidney injury with pyelonephritis, 1 had proteinuria and 1 had had “an elevated serum creatinine.” However, these labs were collected at the time of a symptomatic stone episode and the eGFR calculated for each patient may not be an accurate reflection of these children's true baseline eGFR. Hypercalciuria, hyperoxaluria, and hypocitraturia were identified in 20, 33, and 2% of children, respectively. There were no significant correlations of height *z* score with eGFR, serum calcium or serum bicarbonate; there was a trend toward correlation between increasing height *z*-score and increasing serum calcium levels but did not reach significance (*p* = 0.167) (Figure 1). eGFR significantly correlated with the serum BUN/creatinine ratio, but not the serum bicarbonate (Figure 2). The correlation between dietary markers of protein intake and eGFR, BUN, and BUN/creatinine ratios are presented in Figure 3.

**Table 4:** Percent of patients with metabolic findings.

	**Female**		**Male**		**Statistical test**	***p*-value**
	**Patient # with finding/# with data on results available**	**%**	**Patient # with finding/# with data on results available**	**%**		
Height z-score < −1	11/49	22	2/27	7	Fisher exact	0.12
Bicarbonate < 22 meq/dl	11/58	19	12/43	28	Chi square	0.29
Calcium >10.5 mg/dl	1/60	2	0/42	0	Fisher exact	1.00
Vitamin D >50 ng/ml	0/10	0	1/13	8	Fisher exact	1.00
GFR < 90	16/41	39	7/24	29	Chi square	0.46
GFR>140	4/41	10	2/24	8	Fisher exact	1.00
Hypercalciuria (>4 mg/kg/day)	5/30	17	6/24	25	Fisher exact	0.51
Hyperoxaluria (>40 mg/1.73 m^2^/day)	7/30	23	11/24	46	Fisher exact	0.15
Hypocitraturia (< 180 mg/1.73 m^2^/day)	1/30	3	0/24	0	Chi square	1.00

### Management and Outcomes

The clinical course, procedures, and treatment of pediatric stone patients is presented in Table 5. Males and females had similar rates of hospitalizations, multiple stone episodes, multiple ED visits, and procedures. Pharmacological prevention of stones was only provided to a minority of patients, with citrate or thiazides each prescribed to ≤ 7% of males or females. Dietary recommendations were documented as presented to just under of both male and female patients. Increased citrate intake was recommended to significantly more females than male patients (*p* = 0.01).

**Table 5:** Clinical course, procedures, and treatment.

**Outcome**	**Female (*****n*** **=** **82)**		**Male (*****n*** **=** **54)**		***P*-value**
	**Number**	**%**	**Number**	**%**	
>1 stone episodes	19	23	11	20	0.69
Hospitalization	29	35	19	35	1.00
Multiple ED visits	14	17	14	26	0.21
≥1 procedure	17	21	8	15	0.38
Stent placement	8	10	1	2	0.09
Pyelogram	8	10	3	6	0.52
Lithotripsy	9	11	6	11	1.00
Ureteroscopy	9	11	3	6	0.36
Basket	4	5	1	2	0.35
Tamulosin prescription	38	46	22	41	0.60
Hydrochlorothiazide prescription	2	2	3	6	0.39
Citrate prescription	6	7	4	7	1.00
Dietary recommendations	55	67	40	74	0.38
↑ Fluids	55	67	39	72	0.52
↑ Dietary citrate	17	21	2	4	*0.01
↑ Dietary potassium	2	2	1	2	1.00
↓ Dietary sodium	22	27	15	28	0.92
↓ Dietary oxalate	3	4	4	7	0.43

* *Precedes a significant p value*;

↑ *denotes increasing*;

↓ *denotes decreasing. Significant p-values are indicated in Bold*.

## Discussion

Urinary stone disease is increasing in children, and occurs with a female to male excess of 2.3:1 (8). Female preponderance among pediatric stone formers is distinct from adults and is driven by poorly understood mechanisms. The primary object of this paper was to determine if female pediatric urinary stone formers had different clinical phenotypes compared to males. Based upon our review of the literature, this has not previously been directly measured. Males and females in our population had similar stone risk profiles including urine oxalate, calcium, and citrate levels. Family history of kidney stones was also similar between groups. We did identify sex specific differences between male and female stone patients, some of which were novel. Specifically, female pediatric stone formers were older, had lower height *z*-scores, were more likely to present with flank pain, have positive leukocyte esterase in their urine and have lower mean serum BUN/creatinine ratios. Although there was no significant differences in kidney function between males and females, we identified 9% of patients had eGFR measurements consistent with glomerular hyperfiltration and that increasing eGFR significantly correlates with increasing BUN/creatinine ratios.

Several findings of this study were consistent with prior literature including that females were significantly older than males; this confirmed the high rates of urinary stone formation in female adolescents as described by Dwyer et al. (18) and Novak et al. (8). We also identified that ~65% of stone forming children had a family history of urinary stones, consistent with Spivacow who demonstrated positive family history in 75% of affected children (7). However, very few patients were diagnosed with monogenic stone disease or diseases which predispose to stones such as RTA. These low rates are likely in part due to the paucity of patients evaluated by nephrology and the few patients who completed full metabolic work ups. In previous pediatric studies 17–21% of stone forming children were found to have a monogenic cause for stones. We speculate that a larger percentage of our patients would have been diagnosed with monogenic stones if a greater number of metabolic evaluations were completed and genetic screening incorporated (19).

There were no significant differences between females and males for common stone risk factors including urine calcium, oxalate, citrate, calcium oxalate supersaturation, and calcium phosphate supersaturation; this indicates that an unknown risk factor is responsible for the female excess of pediatric urinary stones.

We identified the novel and unexpected finding that females had lower height *z*-scores (medians 0.2) than males (median 0.8). Why stone forming females have lower height *z*-scores is unclear but the transition of male predominance to female predominance in stone forming children at the onset of puberty is intriguing. Puberty is associated with increases in sex hormones (estrogens and androgens), as well as changing growth hormone (GH) and insulin-like growth factor-1 (IGF-1) levels; together this neuro-endocrine axis controls linear growth, accumulation of muscle mass, and bone mineralization (20). Previous studies have also linked sex hormones to urinary citrate and calcium excretion. Dey et al. (21) demonstrated that adult stone forming women had alterations in urinary citrate and calcium excretion when they reach menopause, and estrogen replacement therapy increases citrate in adult female stone formers (22, 23). We speculate that another possible explanation for lower female height *z*-score includes the number of stone formers with identified chronic diseases that could also affect height including Crohn's disease and CKD. Only one patient was formally diagnosed with renal tubular acidosis (RTA), however, given the lack of standardized work up in this cohort the existence of renal tubular acidosis (RTA) or incomplete RTA in the female patients could contribute to lower height *z* scores (24). The mean serum bicarbonate in these patients was ~24 mEq/L which is toward the lower end of the normal range of 23–29 mEq/L. However, on additional analysis the height *z*-score did not correlate with the serum bicarbonate in either males or female and the mean serum CO_2_ was not lower in female vs. male patients. Nonetheless 12/59 (21%) and 11/43 (26%) of female and male patients, respectively, had a serum CO_2_ < 22 raising the possibility that some type of mild acidosis is present in our cohort. Should our findings be confirmed, additional studies should evaluate renal acidification defects and growth rates. How sex, puberty, and growth all interconnect is not clear and may be linked to renal acidification defects; it is certainly an area deserving of increased research in children and adults.

Our pediatric stone forming cohort also had a relatively high incidence of skeletal fracture, with 15% of males and 12% of females having a documented skeletal fracture. On review of secondary diagnoses we did find several diagnoses that can be associated with skeletal fractures. For females this included three patients with CKD, renal transplant, and Topiramate use, respectively. Among stone forming males with fractures four had comorbidities including one with an extensive medical history of chromosomal abnormality and Evan's syndrome, another with polyarticular juvenile rheumatoid arthritis, a third with arthritis and heavy steroid use and a fourth with ulcerative colitis and heavy steroid use. Once these patients were excluded there remained 8.5% of females and 7.4% of males with skeletal fracture who did not have any co-morbid conditions associated with fracture; these findings are consistent with previous reports of bone mineral density loss in adults and children with stones as well as increased skeletal fracture rates (25, 26). However, one could also argue that children whose chronic diseases which predisposed them to bone disease also predisposed them to high rates of kidney stones formation similar to a previously identified pediatric cohort which showed non-ambulation and use of lithogenic medications to be frequent risk factors for concurrent stone and bone disease (27).

Male urinary stone patients had a significantly higher BUN/creatinine ratio than female patients which might indicate that volume depletion and/or high protein intake is more pronounced in boys. In support of this finding urine specific gravity trended higher in male patients (*p* = 0.07). Low urine volumes associated with poor hydration have previously been reported to correlate with increased urine crystal supersaturation and stone formation, but to our knowledge this study represents the first sex based difference in hydration markers in a pediatric urinary stone patient population (28). Although, the mean serum estimated glomerular filtration rates (eGFRs) were within the normal range of 90–120 ml/min/1.73 m^2^, one of the more concerning findings in our studies was that 35 patients had low eGFR (<90 ml/min/1.73 m^2^) and 9% had a eGFRs >140 m/min/1.73 m^2^ consistent with hyperfiltration. In our cohort the most likely etiologies for this are high protein intake and/or chronic dehydration, both which have been linked to hyperfiltration and urinary stone disease (29–32). Ten of the patients with a low eGFR had a concurrent diagnosis of CKD, pyelonephritis, AKI, proteinuria, cystinuria and/or an elevated creatinine. In addition, labs may have been obtained in the ED during episodes of acute renal colic where patients are at risk for AKI from dehydration and Non-steroidal anti-inflammatory (NSAID) use. Due to the association of CKD and urinary stones in adults (33, 34), it will be important to estimate eGFR, even if the creatinine is in the “normal” range, and follow-up for longitudinal eGFR monitoring. Also noteworthy is that patient's increasing BUN/creatinine ratios significantly correlated with increasing eGFRs. While the BUN/creatinine ratio is typically preserved during CKD progression and intrinsic AKI, an increasing BUN/creatinine ratio indicates that the plasma urea is increased relative to creatinine and is usually seen in the setting of high protein dietary intake, catabolic states, gastrointestinal bleeds, or decreased renal perfusion (35). A similar correlation between the BUN/creatinine ratio and eGFR was noted in a survey of children seen as outpatients in a Pediatric Nephrology clinic; they noted a higher BUN/creatinine ratio in females where ins our stone cohort males had a higher BUN/creatinine ratio (36). Other markers for dietary protein intake including urine urea nitrogen (UUN) and urine sulfate significantly correlated with eGFR supporting a potential link between dietary protein intake and hyperfiltration.

We also identified that female urinary stone patients were >8 times more likely to have leukocyte esterase (LE) on urinalysis when compared to male urinary stone patients. We know that LE is a non-specific marker of white cells within the urine, most often associated with urinary tract infections (UTIs). In Taiwan, 44% of female urinary stone patients had a UTI history (37). Urine cultures were not available and we were unable to differentiate between an inflammatory or infectious state in these patients. Urinary tract infections can occur as result of urinary stones but also are a potential cause of stones (1). Pyelonephritis was listed as a diagnosis for 6 females and 2 males in our cohort, 12 females had a history of UTI and one had a history of urosepsis. Whether the high rate of pyuria is the result of acute UTIs, a more chronic dysbiosis or inflammation from crystalluria remains to be determined.

We were able to identify pediatric urinary stone practice patterns at a single institution level. We noted management similarities and difference between male and female patients. 24 h urine studies were not collected in all patients, and this may reflect the preponderance of referrals to urology who traditionally do fewer urinary assessments than the nephrology group. However, for those patients that did have 24 h urine studies we did not find difference between males and females rates of low urine citrate, hypercalciuria, hyperoxaluria, or low urine volume. CTs were obtained on 67% of male patients and 57% of female patients, making limiting future radiation exposure a priority (38). Nephrocalcinosis was noted in two patients who were diagnosed with RTA and hypercalciuria respectively; this was consistent with previous pediatric studies (39, 40). Most patients had a basic metabolic profile with calcium measured. However, phosphorous, magnesium, parathyroid hormone, vitamin D, and uric acid were obtained in <25% of patients limiting practitioners' ability to diagnose rare disorders. Despite a high risk of recurrent stones, <13% of patients were treated with citrate and/or a thiazide diuretic to help prevent future stones. Finally, no patients had a bone density measured by dual energy X-ray absorptiometry (DXA) despite a fracture history in 12% of the patients, and the known association of urinary stones and low bone density (25, 41, 42). Indications for pharmacologic management and bone mineral density screening of pediatric urinary stone patients should be defined and would benefit from future randomized controlled trials.

Limitations of this study include retrospective design and that data collection was from a single tertiary care pediatric hospital in the Midwest, and thus may limit generalizability. Furthermore, the study lacks protocol standardization and during the study period local nephrology and urology groups differed greatly in their evaluation and treatment patterns; thus only 20% of patients were evaluated by a nephrologist resulting in few patients with a complete metabolic work up and few monogenic stone formers were identified. This center now has a multidisciplinary kidney stone clinic with standardized evaluation protocol. This dataset will serve as a baseline for future studies demonstrating differences in patient outcomes with and without protocol standardization that incorporates evaluation by a pediatric nephrologist. Additionally, we have added a sub-analysis (see Figures 1, 2) of some of the key findings (low height *z*-score and high BUN/creatinine ratio) limited to patients that had components of the urinary stone evaluation completed to identify potential associations. Due to the retrospective nature of the study additional limitations include our reliance on diagnostic codes recorded for billing to identify stone formers and thus may have underestimated the true prevalence of stones. Similarly our retrospective data collection information ensured that data concerning patient ethnicity was not available and made advanced analysis of the pyuria observed in our cohort impossible. Urinary tract infections can be very difficult to diagnose correctly in retrospective work due to frequent lack of urine culture in clinical practice; thus correctly connecting symptoms with urinalysis results to true infection without the definitive assistance of a culture can be very difficult. Further, to truly evaluate the meaning of a positive LE on urinalysis we would also need urine microbiome results due to the expanding body of research demonstrating the clinical importance of the urine microbiome. Individual's urine microbiome contains many bacteria not culturable with standard clinical urine culture techniques and thus a formal evaluation for “sterile” (culture negative) pyuria—which is beyond the scope of this study- would be necessary. Thus, we are limited to reporting that pyuria is significantly more frequent in female vs. male pediatric stone formers. Almost all of our patients were within the World Health Organization definition of the adolescent age range of 10–19 years (43), thus our findings might not be applicable to younger children. Our small sample size relegates many of our findings to hypothesis generating. However, this is the largest study to evaluate stone forming children for sex specific characteristics published to date that we were able to find.

In summary, this study provides preliminary evidence of female vs. male specific differences in pediatric urinary stone presentation, management, laboratory results, and outcomes. Based on our results, follow-up studies to validate differential height *z*-scores, increased urine LE in female urinary stone patients, and increased rates of volume depletion markers in male urinary stone patients are needed. We identified that an increasing BUN/creatinine ratio is associated with increasing eGFR and 9% of patients have labs consistent with glomerular hyperfiltration. A high protein diet and/or chronic hypohydration may be a mechanism, with an origin in the pediatric age range, for the association between CKD and urinary stone disease. Additionally, pediatric urinary stone management would benefit from guidelines regarding the indications for CT to limit radiation exposure, when DXA scans are indicated, and consensus on when to initiate pharmacological therapy.

## Author Contributions

All authors approved the final version of the manuscript. AS and KK assisted in the conception of the project, data collection, and data analysis. They were instrumental in drafting the paper and provided intellectual content for the manuscript. RR, SM, and FS assisted in data analysis and interpretation, and revising the manuscript. AK was involved in designing the project, collecting data, and drafting the manuscript.

### Conflict of Interest Statement

The authors declare that the research was conducted in the absence of any commercial or financial relationships that could be construed as a potential conflict of interest.
